# Association between Mastitis Occurrence in Dairy Cows and Bedding Characteristics of Compost-Bedded Pack Barns

**DOI:** 10.3390/pathogens12040583

**Published:** 2023-04-12

**Authors:** Gustavo Freu, Breno Luis Nery Garcia, Tiago Tomazi, Gabriela Siqueira Di Leo, Larissa Schneider Gheller, Valerio Bronzo, Paolo Moroni, Marcos Veiga Dos Santos

**Affiliations:** 1 Department of Animal Nutrition and Production, School of Veterinary Medicine and Animal Science, University of São Paulo, Pirassununga 13635-900, São Paulo, Brazil; gustavofreu@usp.br (G.F.); breno.luis.garcia@usp.br (B.L.N.G.);; 2 Department of Veterinary Medicine and Animal Sciences—DIVAS, University of Milan, 26900 Lodi, Italypaolo.moroni@unimi.it (P.M.); 3 Laboratorio di Malattie Infettive degli Animali—MiLab, University of Milan, 26900 Lodi, Italy; 4 Ruminant Technical Services, Merck Animal Health, Kenilworth, NJ 07033, USA; 5 Department of Animal Science, School of Animal Science and Food Engineering, University of São Paulo, Pirassununga 13635-900, São Paulo, Brazil; 6 Quality Milk Production Services, Animal Health Diagnostic Center, Cornell University, Ithaca, NY 14853, USA

**Keywords:** compost barn, clinical mastitis, subclinical mastitis, dairy cows, bedding characteristics

## Abstract

Compost-bedded pack barns (CB) are receiving increasing attention as a housing system that can potentially improve the welfare of dairy cows. This study characterized the frequency and profile of pathogens isolated from clinical (CM) and subclinical (SCM) mastitis in dairy cows housed in CB. It evaluated the association between mastitis occurrence and bedding characteristics in CB systems. Over six months, seven dairy herds were visited monthly for milk and bedding sample collections. Milk samples from mastitis cases were submitted to microbiological identification by matrix-assisted laser desorption ionization-time of flight (MALDI-TOF MS). Bedding samples were submitted to physical-chemical (pH, organic matter, moisture, and carbon to nitrogen ratio) and microbiological counting (total bacterial counts, coliforms, streptococci, and staphylococci) analyses. Regression analysis was used to determine the association between mastitis occurrence and CB characteristics. Our results showed that *Escherichia coli* and environmental streptococci were the most frequently isolated pathogens from CM cases, while *Staphylococcus chromogenes* and contagious pathogens (*Staphylococcus aureus* and *Streptococcus agalactiae*) were the most commonly isolated from SCM cases. Bedding moisture content was positively associated with the incidence of CM. The bedding carbon to nitrogen ratio was negatively associated with the incidence of SCM, and the bedding total bacteria counts tended to be associated with the incidence of SCM. Bedding counts of coliforms positively associated with the prevalence of SCM. Our results can support decision-makers in the dairy industry seeking strategies for bedding management and mastitis control.

## 1. Introduction

Compost-bedded pack barns, also known as compost dairy barns (CB), are housing systems for dairy cows that consist of an open area without partitions where cows have free access to the bedding and feeding alley areas. CB systems use organic material as a substrate that remains in constant composting activity [1], providing a dry and comfortable surface for dairy cows [2,3,4]. This has made CB an attractive system for milk production, so much so that CB facilities have already been described in North America, Europe, and Brazil [5,6,7,8]. However, despite some benefits, the CB system may present a higher risk of mastitis when poorly managed.

Bovine mastitis is a frequent disease in dairy herds which is commonly related to infection by pathogenic bacteria and related to significant economic losses to the dairy industry [9,10]. Coliforms (including *Escherichia* spp., *Klebsiella* spp., and other Gram-negative bacteria) and environmental streptococci (e.g., *Streptococcus uberis* and *Streptococcus dysgalactiae*) are the most prevalent pathogens causing clinical mastitis (CM) on many modern dairy farms that have successfully controlled contagious mastitis [11,12,13]. Additionally, non-*aureus* staphylococci and environmental streptococci are described as one of the pathogens groups most isolated from subclinical mastitis (SCM) cases worldwide [14,15]. Bedding was described as an important source of udder exposure to environmental mastitis pathogens [16]. Therefore, to better control environmental mastitis in CB systems, we need an improved understanding of the relationships between bedding characteristics and mastitis occurrence.

Previous studies reported failures in the bedding composting process management, such as suboptimal bedding temperature (<55 °C; [5]), bedding compaction [17], and excessive moisture (>60%; [18]). Bedding moisture has been reported as one of the most challenging characteristics to control in CB systems because it can be influenced by bedding management and weather conditions [2]. With uncontrolled moisture, bedding material can adhere more easily to the mammary gland, increasing the risk of mastitis caused by environmental coliforms and streptococci [17]. According to Fávero et al. [17], the risk of environmental CM increased by 5.7%, adding a one-unit percentage in bedding moisture content.

As one of the main objectives of CB management is to keep the composting process active by providing adequate conditions for microbial multiplication, bacterial counts in these systems are high [2], which can be a risk factor for udder health. Bedding material has been described as a primary source of bacterial contamination of the mammary gland [19], and different bedding materials were shown to support the growth of mastitis-causing pathogens [20,21]. In this sense, teat skin contamination and the risk of new intramammary infections can be influenced by the type and physical-chemical characteristics of the bedding material. Therefore, poor bedding management conditions can contribute to undesirable composting features (e.g., high moisture content and compacted areas), contribute to bedding material adherence on the udder surface, and increase the cow’s risk of developing SCM or CM [1].

Results from field studies can be useful to better understand bedding characteristics that need attention and can support decision-makers in the dairy industry seeking strategies for bedding management and mastitis control. Thus, this study aimed to evaluate (a) the frequency and profile of mastitis-causing pathogens in cows housed in CB; and (b) the association between mastitis occurrence (CM and SCM) and physical-chemical and microbiological characteristics of bedding material in CB systems.

## 2. Materials and Methods

### 2.1. Herd Selection and Study Protocols

A longitudinal study was conducted using seven dairy herds in São Paulo state, Brazil, based on convenience sampling, according to the availability and interest level of dairy farmer participation in the study and proximity to the university (<150 km). Dairy herds were visited monthly for six months, from December 2018 to May 2019, for data and sample collection. Specific criteria for selection of herds included: (a) lactating cows housed in the CB system; (b) thorough records of individual data (e.g., days in milk, parity, milk production) of all lactating cows in the herd; and (c) dairy herd improvement (DHI) participation.

Before beginning the study, herds were visited and presented with the objectives of the study and collaborator training to demonstrate the correct identification of mastitis, classification of severity scores, and milk sample collection from CM cases. CM severity was recorded as mild, moderate, and severe, according to Wenz et al. [22]. Each farm had assigned farm personnel who were responsible for collecting milk samples from CM cases and recording the data (e.g., cow, affected mammary quarter, and severity score). During the study, kits with materials for milk sample collection (gauzes, 70% iodized alcohol, and sterile tubes) were provided to each herd.

### 2.2. Farm and Cow Characteristics

The bedding area available to cows was measured to calculate the stocking density (m^2^/cow). Stocking density calculations were obtained by dividing the total bedding area by the number of lactating cows using the bedding. Additional information related to bedding management (e.g., bedding type and tilling frequency) was recorded. Except for one (Herd E), all other herds had free access to concrete feeding areas separate from the bedding areas. All herds had fans installed over the bedding area, but Herd E farm management kept fans off during the study period. All herds were milked in herringbone pit parlors, and the milking routine included CM diagnosis (e.g., examination of the first milk streams in a streak cup) and use of pre- and post-milking teat dip.

Holstein (*n* = 587), Girolando (*n* = 586; crossbred *Bos taurus* × *Bos taurus indicus*), and Gir (*n* = 22) dairy cows were included in the study. Cows were sampled monthly to collect milk samples for somatic cell counts (SCC) and microbiological culture. All lactating cows were kept in the CB system and fed according to the nutritional management of each herd.

### 2.3. Milk and Bedding Sample Collection

Before each visit and as part of the herds’ monthly routine (DHI participation), composite milk samples were collected from all lactating cows in a 50-mL plastic tube containing 2-bromo-2-nitropropane-1,3-diol chemical preservative (Bronopol, Microtabs II, D & F Control Systems Inc., Norwood, MA, USA) for SCC analyses. Composite milk samples for SCC (approximately 40 mL) were collected from the milk meters at the end milking time of each cow. Cows with >200,000 SCC/mL had composite milk samples collected and submitted to microbiological identification by matrix-assisted laser desorption ionization time of flight (MALDI-TOF MS). Cows with CM had milk samples collected from the affected mammary quarter. Samples from CM cases between farm visits were frozen (−20 °C) until the next visit. Milk sample collection procedures were performed according to the National Mastitis Council guidelines [23].

Bedding samples were collected monthly from each herd for physical-chemical and microbiological analyses. The bedding area was divided into 12 equal squares, as described by Barberg et al. [24], and using a polychloride vinyl pipe, a representative sample from the superficial (±10 cm) and deep layer (±20 cm) was collected from each square. These samples were collected while cows were being milked and before bedding tilling. In addition, bedding temperature was measured at both depths of each square using a digital thermometer (Incoterm, Porto Alegre, Brazil).

The bedding samples were mixed to obtain a homogeneous and representative sample of each studied layer (i.e., superficial and deep), and the samples were frozen until the microbiological analyses. Mixing both collected layers, a representative sample of the entire bedding area was obtained for physical-chemical studies [17].

### 2.4. Milk and Bedding Analysis

SCC was analyzed by flow cytometry using the Somacount 300^®^ equipment (Bentley Instruments Inc., Chaska, MN, USA).

Microbiological identification of mastitis-causing pathogens was performed by MALDI-TOF MS. An aliquot of milk (10 µL) was inoculated onto a blood agar plate, supplemented with 5% bovine blood, and incubated at 37 °C for 24–48 h. Obtained bacterial isolates were submitted for identification by MALDI-TOF MS, according to Barcelos et al. [25]. It was considered as species-level identification (MALDI score ≥ 2), genus-level (>1.7 and <2), and no reliable identification (<1.7). For non-*aureus* staphylococci species identification, a cutoff score ≥ 1.7 was considered [26]. Contaminated was defined as growth of three or more distinct microorganisms in the same milk sample.

For bedding samples, physical-chemical analyses [moisture (%), organic matter (%), carbon-nitrogen ratio (C/N), and pH] were performed according to official methods [27]. Microbiological analyses were performed according to Zdanowicz et al. [28]. Briefly, 10 g of a bedding sample was diluted in 90 mL of peptone water (0.1%), followed by serial dilutions (10^−1^ to 10^−6^). The blood agar (Oxoid, Basingstoke, UK), McConkey (KASVI, São José dos Pinhais, Brazil), Edward’s modified media (Oxoid, Basingstoke, UK), and Vogel Johnson (Acumedia, Lansing, MI, USA) were used for the total bacterial count, coliforms, streptococci, and staphylococci, respectively. Each culture media was prepared and interpreted according to the manufacturer’s recommendations. For all microbiological analyses, 100 μL of the inoculum was added to the center of each plate and spread over the entire plate surface. Bedding bacteria counting was performed manually. Plates with visible signs of contamination (e.g., mold on the agar surface) were discarded, and a new analysis was performed.

### 2.5. Data Analyses

Data were recorded in Excel spreadsheets (Microsoft Office, 2016) and verified before statistical analysis. Data analysis was performed using statistical software SAS version 9.4 (SAS Institute, Cary, NC, USA). Before analyses, data were screened for residual normality, and the microbiological count values were log-transformed to meet this criterion. The results of microbial counts were expressed as log_10_ cfu/g. Descriptive analyses were performed to describe herd and bedding characteristics and the frequency of mastitis-causing pathogens. Linear mixed models with repeated measures were constructed using PROC MIXED to determine the association between mastitis occurrence and physical-chemical and microbiological characteristics of bedding material. The farm visit was considered as the experimental unit.

Cows were considered to have SCM when presenting >200,000 SCC/mL [29], and CM cases were defined as a visual alteration of the milk, with or without local or systemic signs of infection [30]. For cows that experienced repeated episodes of CM, only cases 14 days after a previous case were considered new cases [17]. Indexes of mastitis prevalence and incidence were evaluated, as described by Fávero et al. [17]. During the experimental period, two herds did not record the CM cases and were excluded from the CM incidence analysis. Furthermore, SCC data were unavailable in the month before the beginning of the study, which did not allow for the estimation of the incidence of SCM in the first month of the study.

Explanatory mastitis prevalence and incidence variables were bedding physical-chemical (moisture, organic matter, C/N, and pH) and microbiological characteristics (total bacterial count, coliforms, streptococci, and staphylococci). Preliminary, univariate linear regression was used to identify unconditional relationships between explanatory variables and study outcomes. Only variables with *p* < 0.20 were included and evaluated in stepwise model selection to select the final models [16]. Only variables with *p* ≤ 0.10 were kept in the final model. The farm was offered to the model for all analyses as a random effect. Statistical significance was declared when *p* < 0.05, and the tendency to significance was considered if the *p*-value was >0.05 and <0.10.

## 3. Results

### 3.1. Farm and Cow Characteristics

This study’s average stocking density was 11.8 (8.3 to 16.0; Table 1). Bedding material was tilled at least twice daily between milkings (2.4 ± 0.5; mean ± SD) for all herds. All herds were housed using sawdust as bedding material (Table 2).

The mean of lactating cows was 111.7 (ranging from 45 to 208), with a mean daily production of 29.0 (±4.0) L/cow/day (Table 1). The overall mean of bulk milk tank SCC was approximately 391.4 × 10^3^ (±188.5) cells/mL.

### 3.2. Frequency of Mastitis-Causing Pathogens

A total of 272 CM cases were recorded during the study period (Table 2 and Table 3). Of this total, 43.0% (*n* = 117) of CM cases had negative culture results (i.e., no microbiological growth). Among the positive cultures, environmental pathogens were the most frequent. Of these, *Escherichia coli* (*n* = 34; 12.5% of the total samples) was the most frequent pathogen, followed by *Streptococcus dysgalactiae* (*n* = 22; 8.1%) and *Streptococcus uberis* (*n* = 19; 7.0%). Concerning the distribution of CM severity scores, 50.7% (138/272) were mild; 29.0% (79/272) were moderate; 8.5% (23/272) severe; and 11.8% (32/272) had no severity recorded.

Regarding SCM, with 1563 milk samples analyzed, 40.2% (*n* = 629) had negative culture results (Table 4). *Staphylococcus chromogenes* (*n* = 389; 24.9% of the total samples), *Streptococcus agalactiae* (*n* = 84; 5.4%), and *Staphylococcus aureus* (*n* = 64; 4.1%) were the most frequently found Gram-positive pathogens. *Escherichia coli* (*n* = 11; 0.7%) was the most prevalent microorganism among Gram-negative bacteria.

### 3.3. Bedding Characteristics and Mastitis Indexes

**Figure 1:**
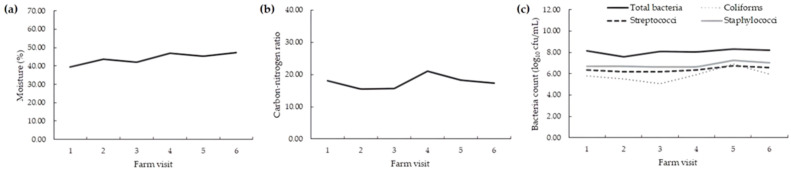
Monthly physical-chemical (**a**,**b**) and microbiological (**c**) characteristics of bedding material from seven dairy herds housed in compost barn systems.

Bedding moisture content was 44.2% ± 8.3 (ranging from 30.4 to 61.9%; Table 5). The mean pH, organic matter, and C/N ratio were 8.3 ± 0.5, 55.7 ± 11.6, and 17.6 ± 7.8, respectively. The total bacterial count in the surface layer was 8.1 (±0.5 log_10_ cfu/g). Coliforms, streptococci, and staphylococci counts ranged from 5.9 to 6.8 log_10_ cfu/g (Table 5; Figure 1).

**Figure 2:**
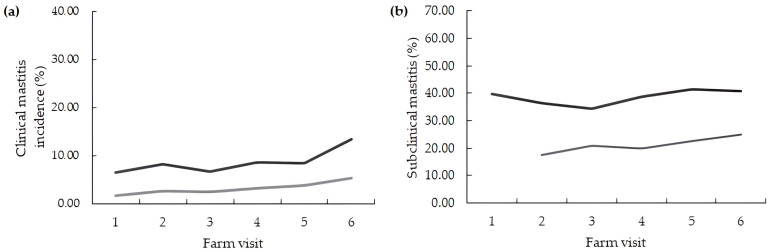
Monthly mastitis indexes from seven dairy herds housed in compost barn systems: (**a**) incidence of clinical mastitis by all pathogens (dark line) and by environmental pathogens (light line); (**b**) prevalence (dark line) and incidence (light line) of subclinical mastitis according to the farm visit, respectively.

The incidence of CM for all pathogens was 8.9 (±3.6), and this incidence was 3.4 (±2.9) when considering only environmental pathogens (Table 5; Figure 2). The incidence of SCM was 21.2 (±9.6), and the prevalence of SCM was 38.7 (ranging from 23.0 to 56.0).

Bedding moisture content (*p* = 0.004) and counting of staphylococci (*p* = 0.001) were unconditionally associated with the incidence of CM for all pathogens (Table 6). Still, bedding moisture (*p* = 0.004) remained the sole predictor in the final multivariate model (Table 7). Moisture content was the only predictor associated (*p* = 0.005) with the incidence of environmental CM in univariate and multivariate analyses (Table 6 and Table 7).

The bedding C/N ratio was negatively associated (*p* = 0.037) with the incidence of SCM, and the counting of total bacteria tended to be associated (*p* = 0.055) with the incidence of SCM in the final model (Table 7). Bedding counting of coliforms was positively related to the prevalence of SCM (*p* = 0.026) in both univariate and multivariate analyses (Table 6 and Table 7).

## 4. Discussion

Compost dairy barns have received increased interest as a housing system for dairy cows, mainly because they can improve animal welfare [4]. However, the performance of CB systems concerning udder health largely depends on bedding management. This study describes the mastitis-causing pathogens profile in dairy cows confined in CB. It provides an approach to the physical-chemical and microbiological characteristics of bedding that need farm-level attention for mastitis control. Our results can support farms seeking improvement in bedding management and mastitis control strategies in their herds.

### 4.1. Frequency and Profile of Mastitis Pathogens

In our study, *Escherichia coli* (12.5% of the total samples) and environmental streptococci (>15.0%) were the most frequent pathogens isolated from CM cases, which agrees with previous reports (11.0% and >15.0%; 6.0% and >10.0%; 22.6% and 12.7% [14,31,32], respectively). Coliforms and environmental streptococci are described in many cow habitats, including bedding materials [11,33]. These microorganisms opportunistically cause mastitis, usually resulting from bacterial migration from the contaminated environment via the teat canal. Therefore, hygiene, pre-milking, and bedding management are keys to reducing the exposure of cows to environmental mastitis.

A total of 43.0% of the CM samples analyzed in our study had negative culture results. These findings are similar to other studies evaluating CM milk samples (32.5 and 44.0% [17,31], respectively). Several factors can influence negative culture results. For example, pathogens with fastidious growth that require special cultivate conditions such as specialized equipment and reagents in laboratory settings (e.g., *Mycoplasma* spp. [34]), sample storage conditions (e.g., sample freezing and increased length of storage [35]), and spontaneous clearance of the pathogen by a cow’s immune system [36,37]. In our study, *Escherichia coli* was the most frequent pathogen isolated from CM cases, and it has been associated with results of no growth in a preview study [38]. Therefore, results of no growth frequency here can be partially attributed to infections caused by *Escherichia coli*, in which spontaneous cure occurred, or because of the on-farm freezing of samples before microbiological analysis.

The frequency of SCM pathogens observed in this study was similar to other studies in which a higher frequency of Gram-positive pathogens was reported (52.9% in our study vs. 48.6% [17], respectively). *Staphylococcus chromogenes* and contagious pathogens (e.g., *Staphylococcus aureus* and *Streptococcus agalactiae*) were our study’s most frequent bacteria isolated from SCM cases. Despite the success of controlling contagious mastitis in many countries [39,40], contagious pathogens are still a problem in Brazil. Evaluating bulk tank milk samples from 306 dairy herds from the south of Minas Gerais state, Brazil, Mesquita et al. [41] reported that almost 50% of herds had problems with both *Staphylococcus aureus* and *Streptococcus agalactiae*. Additionally, contagious pathogens were the most frequently isolated group of bacteria from SCM samples in cows housed in CB systems [17]. Therefore, our results corroborate the importance of herd biosecurity, pre- and post-milking teat disinfection, and reducing the reservoir of infection in herds (e.g., strategic treatment and culling) for controlling contagious pathogens.

Finally, recognizing the herds’ pathogen distribution can help implement control strategies to reduce udder exposure and ensure responsible antimicrobial use.

### 4.2. Bedding Characteristics and Mastitis Indexes

Bedding moisture content was associated with the incidence of CM of all pathogens and the incidence of environmental CM in our study, which agrees with what was previously reported by Fávero et al. [17]. Bedding moisture has been reported as one of the most challenging characteristics to control in CB systems because it can be influenced by bedding management and weather conditions [2]. Wet bedding can dirty the cow’s udder [42], which may increase mastitis risk caused by environmental pathogens [17]. Therefore, bedding management is critical to encouraging microbial activity, minimizing pathogen exposure, and maintaining cow cleanliness [43]. In this sense, maintaining a dry surface for dairy cows can reduce the incidence of CM.

The mean of the bedding C/N ratio in our study was 17.6 ± 7.8, which is similar to results described in CB systems in the Minnesota region (15.5 [43]), although lower than recommended (25:1 to 30:1) to optimize the composting process [44]. We observed that the bedding C/N ratio was negatively associated with the incidence of SCM in this study. Carbon and nitrogen are the primary nutrients microorganisms require during the composting process. Therefore, we can speculate that the C/N ratio in our study was enough for microbial activity in the system but not enough to optimize the composting process to the point of reaching high temperatures for pathogen devitalization (e.g., >54 °C [43,45]). This is supported by the high microbiological counts observed in our study. In agreement with us, Leso et al. [2] report that most mastitis-causing bacteria can grow at the temperatures recorded in composting packs.

In addition, counting total bacteria tended to be positively associated with the incidence of SCM in the multivariate model. As one of the main objectives of CB management is to maintain composting process activity, promoting adequate conditions for microbial multiplication, most of the bacterial counts reported in CB systems are high [2], as reported here (≥5.9 log_10_ cfu/g) and in previous studies (>5.0 log_10_ cfu/g [5,45]). Most mastitis-causing bacteria can survive in CB systems [7] because they thrive in similar conditions to composting bacteria and microbes [5]. In this case, it is challenging to eliminate mastitis-causing bacteria in a composting environment. Therefore, this environment can act as a source of udder contamination. Excellent cow preparation procedures at milking time and effective bedding management are essential for reducing teat skin contamination and mastitis risk in cows housed in CB systems.

Bedding counts of coliforms were positively associated with the prevalence of SCM in both univariate and multivariate analyses, which is not expected as coliforms are usually associated with CM cases [11]. Fávero et al. [17] did not report associations between bedding microbiological analysis and mastitis occurrence. We used SCC to calculate the prevalence of SCM, and contagious pathogens were one of the most frequent groups isolated in our study. This is a limitation of our study because the impact of contagious pathogens on the individual SCC of cows probably resulted in the difficulty of separating the effects of bedding characteristics on mastitis prevalence. Therefore, selecting herds with controlled contagious mastitis will be necessary for future studies. On the other hand, although not evaluated here, we cannot rule out latent infections caused by coliforms [46].

We demonstrated that bedding physical-chemical and microbiological characteristics could affect mastitis occurrence in dairy cows housed in the CB system. Our results could support management decisions about bedding to improve udder health and mastitis control strategies in dairy herds.

## 5. Conclusions

*Escherichia coli* and environmental streptococci were the most frequent pathogens isolated from CM cases. *Staphylococcus chromogenes* and contagious pathogens (*Staphylococcus aureus* and *Streptococcus agalactiae*) were the most frequent causes of SCM in dairy cows housed in CB systems. Bedding moisture content was positively associated with the incidence of CM. The bedding C/N ratio was negatively associated with the incidence of SCM, and the counting of total bacteria tended to be associated with the incidence of SCM. Bedding counts of coliforms were positively associated with the prevalence of SCM. We demonstrated that bedding physical-chemical and microbiological characteristics could affect mastitis occurrence in dairy cows housed in the CB system. Therefore, bedding may be a source of mastitis. Molecular studies should be performed to investigate the epidemiology and the udder infection sources in dairy cows housed in CB.

## Figures and Tables

**Table 1:** Characteristics of seven dairy herds housed in compost barns evaluated over six months.

Variable	N ^1^	Mean	SD ^2^	Min ^3^	Max ^4^
Lactating cows (*n*)	42	111.7	52.3	45.0	208.0
BMSCC ^5^ (1000 scc/mL)	30	391.4	203.5	133.0	816.0
Milk production (L/cow/day)	42	29.0	4.0	21.4	35.7
Stocking density (m^2^/cow)	42	11.8	1.5	8.3	16.0
Bedding tilling (times/day)	42	2.4	0.5	2.0	3.0

^1^ Number of observations; ^2^ standard deviation; ^3^ minimum; ^4^ maximum; ^5^ bulk tank milk somatic cell count.

**Table 2:** Herd characteristics and number of CM and SCM samples from seven dairy herds housed in compost barn evaluated by six months.

Herd *	Lactating Cows ^1^	Cow Milk Yield (L/Day) ^2^	Stocking Density (m^2^/Cow)	Bedding Tilling (Times/Day)	Bedding Samples (*n*) ^3^	CM ^4^ Samples (*n*)	SCC ^5^ Samples (*n*)
A	175	24.0	11.1	2	6	114	1040
B	56	23.7	13.0	3	6	0	337
C	195	32.1	13.0	3	6	0	1163
D	71	27.4	11.8	2	6	24	427
E	132	33.3	12.8	3	6	68	771
F	78	29.9	11.0	2	6	38	463
G	76	32.7	9.8	2	6	28	424
Total	-	-	-	-	42	272	4625

^1^ Average number of lactating cows; ^2^ average cow milk yield; ^3^ number of bedding samples collected for physical-chemical and microbiological analyses; ^4^ number of clinical mastitis samples; ^5^ number of milk samples for somatic cell counts analyses. * Data from six months of evaluation.

**Table 3:** Frequency and profile of pathogens isolated from CM cases (*n* = 272) in dairy cows housed in compost barns.

Pathogens	*n*	%
No growth	117	43.01
Gram-positive		
*Strep. dysgalactiae*	22	8.09
*Strep. uberis*	19	6.99
Non-*aureus* staphylococci		
*Staph. chromogenes*	17	6.25
*Staph. haemolyticus*	3	1.10
*Staph. hyicus*	1	0.37
*Staph. pasteuri*	1	0.37
*Staph. simulans*	1	0.37
*Staph. xylosus*	1	0.37
*Staph. aureus*	9	3.30
*Corynebacterium* spp.	6	2.20
*Strep. gallolyticus*	4	1.47
*Enterococcus faecalis*	2	0.73
*Strep. agalactiae*	2	0.73
*Strep. canis*	2	0.73
*Corynebacterium bovis*	1	0.37
*Strep. alactolyticus*	1	0.37
*Enterococcus faecium*	1	0.37
*Lysinibacillus boronitolerans*	1	0.37
*Strep. pluranimalium*	1	0.37
Gram-negative		
*Escherichia coli*	34	12.50
*Klebsiella pneumoniae*	14	5.15
*Serratia marcescens*	2	0.73
*Enterobacter cloacae*	1	0.37
*Paenibacillus cookii*	1	0.37
*Pseudomonas aeruginosa*	1	0.37
*Stenotrophomonas maltophilia*	1	0.37
Others		
*Candida tropicalis*	2	0.73
*Candida kefyr*	1	0.37
*Candida rugosa*	1	0.37
*Prototheca* spp.	1	0.37
*Contaminated* ^1^	1	0.37
Total	272	100.00

^1^ Contaminated > 2 pathogens isolated in the same milk sample.

**Table 4:** Frequency and profile of pathogens isolated from SCM cases (*n* = 1563) in dairy cows housed in compost barns.

Pathogens	*n*	%
No growth	629	40.24
Gram-positive		
Non-*aureus* staphylococci		
*Staph. chromogenes*	389	24.89
*Staph. simulans*	39	2.50
*Staph. hyicus*	20	1.28
*Staph. haemolyticus*	12	0.77
*Staph. xylosus*	5	0.32
*Staph. saprophyticus*	3	0.19
*Staph. capitis*	2	0.13
*Staph. auricularis*	1	0.06
*Staph. epidermidis*	1	0.06
*Staph. hominis*	1	0.06
*Staph. sciuri*	1	0.06
*Staph. warnieri*	1	0.06
*Staph.* spp. ^1^	5	0.32
*Strep. agalactiae*	84	5.38
*Staph. aureus*	64	4.09
*Corynebacterium bovis*	45	2.88
*Strep. uberis*	44	2.82
*Strep. dysgalactiae*	31	1.98
*Corynebacterium* spp.	21	1.34
*Lactococcus garvieae*	10	0.64
*Lactococcus lactis*	9	0.58
Other Gram-positive ^2^	39	2.50
Gram-negative		
*Escherichia coli*	11	0.70
*Klebsiella pneumoniae*	8	0.51
*Serratia marcescens*	6	0.38
*Pseudomonas aeruginosa*	3	0.19
*Klebsiella variicola*	2	0.13
*Pseudomonas* spp.	1	0.06
*Serratia aureylitica*	1	0.06
Other Gram-negative ^3^	21	1.34
Others		
Mixed culture ^4^	39	2.50
*Prototheca* spp.	7	0.45
*Candida rugosa*	3	0.19
*Candida kefyr*	2	0.13
*Candida krusei*	1	0.06
*Candida parapsilosis*	1	0.06
Contaminated ^5^	1	0.06
Total	1563	100.00

^1^ Staphylococci not identified at species-level by MALDI-TOF MS; ^2^ *Streptococcus gallolyticus* (*n* = 6), *Aerococcus viridans*, *Corynebacterium efficiens* and *Paenibacillus lactis* (*n* = 3 of each species), *Enterococcus faecalis*, *Enterococcus faecium*, *Corynebacterium camporealensis*, *Macrococcus caseolyticus*, *Streptococcus lutetiensis* and *Streptococcus parauberis* (*n* = 2 of each species), *Arthrobacter mysorens*, *Arthrobacter polychromogenes*, *Cellumonas flavigena*, *Corynebacterium amycolatum*, *Enterococcus hirae*, *Helcococcus ovis*, *Kocuria salsia*, *Lactobacillus gasseri*, *Micrococcus luteus*, *Micrococcus lylae*, *Streptococcus pluranimalium* and *Trueperella pyogenes* (*n* = 1 of each species); ^3^ *Acinetobacter* spp., *Citrobacter koseri* and *Enterobacter cloacae* (*n* = 4 of each species), *Moraxella osloensis* (*n* = 3), *Pasteurella multocida* (*n* = 2), *Acinetobacter pittii*, *Acinetobacter towneri*, *Acinetobacter ursingii* and *Neisseria subflava* (*n* = 1 of each species); ^4^ milk sample with two different microorganisms isolated in the microbiological culture; ^5^ contaminated > 2 pathogens isolated in the same milk sample.

**Table 5:** Physical-chemical and microbiological characteristics of bedding and mastitis indexes from seven dairy herds housed in compost barn systems.

Variable	N ^1^	Mean	SD ^2^	CV ^3^	Min ^4^	Max ^5^
**Bedding physical-chemical characteristics** ^6^						
Moisture (%)	42	44.2	8.3	18.8	30.4	61.9
pH	42	8.3	0.5	6.1	7.4	9.5
Organic matter (%)	42	55.7	11.6	20.9	33.8	77.1
Carbon-nitrogen ratio	42	17.6	7.8	43.9	9.0	48.0
Temperature (°C)						
Surface	42	35.9	4.9	13.7	26.4	45.6
Deep layer	42	41.4	6.8	16.4	27.7	55.0
**Bedding bacteria counting** ^7^						
Total bacteria (log_10_ cfu/g)	42	8.1	0.5	6.1	6.6	8.8
Coliforms (log_10_ cfu/g)	42	5.9	0.9	15.7	3.3	7.6
Streptococci (log_10_ cfu/g)	42	6.4	0.6	8.7	5.3	7.4
Staphylococci (log_10_ cfu/g)	42	6.8	0.6	8.4	5.0	7.9
**Mastitis indexes**						
Incidence of CM (all pathogens) ^8^	26	8.9	3.6	40.5	4.3	19.5
Incidence of environmental CM ^9^	26	3.4	2.9	84.3	0.0	10.7
Incidence of SCM ^10^	35	21.2	9.6	45.2	3.1	44.4
Prevalence of SCM	42	38.7	7.8	20.1	23.0	56.0

^1^ Number of observations; ^2^ standard deviation; ^3^ coefficient of variation; ^4^ minimum; ^5^ maximum; ^6^ bedding physical-chemical characteristics were estimated on composite samples created by mixing the surface and deep bedding samples; ^7^ superficial bedding layer data (± 10 cm); ^8^ clinical mastitis; ^9^ environmental pathogens: *Candida kefyr*, *Candida rugosa*, *Candida tropicalis*, *Escherichia coli*, *Enterobacter cloacae*, *Enterococcus faecalis*, *Enterococcus faecium*, *Klebsiella pneumoniae*, *Prototheca* spp., *Pseudomonas aeruginosa*, *Salmonella* sp., *Streptococcus dysgalactiae*, *Serratia marcescens*, *Streptococcus canis*, *Streptococcus gallolyticus*, *Streptococcus oralis*, *Streptococcus pluranimalium*, *Streptococcus alactolyticus*, *Streptococcus uberis*; ^10^ subclinical mastitis.

**Table 6:** Unconditional associations (*p* < 0.20) between explanatory variables and study outcomes.

Outcome (Bold Letters) and Explanatory Variables	Estimate	SE ^1^	*p*-Value
**Incidence of CM (all pathogens)** ^2^			
Bedding moisture (%)	0.49	0.14	0.004
Counting of staphylococci (log_10_ cfu/g)	4.46	1.10	0.001
**Incidence of environmental CM** ^3^			
Bedding moisture (%)	0.27	0.09	0.005
Organic matter (%)	−0.12	0.07	0.105
Counting of streptococci (log_10_ cfu/g)	1.40	0.85	0.115
Counting of staphylococci (log_10_ cfu/g)	1.86	1.34	0.178
**Incidence of SCM** ^4^			
Organic matter (%)	−0.23	0.16	0.171
Carbon-nitrogen ratio	−0.56	0.42	0.190
Counting of total bacteria (log_10_ cfu/g)	8.85	5.67	0.130
**Prevalence of SCM**			
Counting of coliforms (log_10_ cfu/g)	2.77	1.20	0.026

^1^ Standard error; ^2^ clinical mastitis; ^3^ environmental pathogens: *Candida kefyr*, *Candida rugosa*, *Candida tropicalis*, *Escherichia coli*, *Enterobacter cloacae*, *Enterococcus faecalis*, *Enterococcus faecium*, *Klebsiella pneumoniae*, *Prototheca* spp., *Pseudomonas aeruginosa*, *Salmonella* sp., *Streptococcus dysgalactiae*, *Serratia marcescens*, *Streptococcus canis*, *Streptococcus gallolyticus*, *Streptococcus oralis*, *Streptococcus pluranimalium*, *Streptococcus alactolyticus*, *Streptococcus uberis*; ^4^ subclinical mastitis.

**Table 7:** Associations between explanatory variables and study outcomes derived from multivariable analyses.

Variable	Estimate	SE ^1^	*p*-Value
**Incidence of CM (all pathogens)** ^2^			
Intercept	−14.23	6.99	
Bedding moisture (%)	0.49	0.14	0.004
**Incidence of environmental CM** ^3^			
Intercept	−9.39	4.20	
Bedding moisture (%)	0.27	0.09	0.005
**Incidence of SCM** ^4^			
Intercept	−49.32	41.49	
Carbon-nitrogen ratio	−0.92	0.42	0.037
Counting of total bacteria (log_10_ cfu/g)	10.53	5.21	0.055
**Prevalence of SCM**			
Intercept	22.98	7.23	
Counting of coliforms (log_10_ cfu/g)	2.77	1.20	0.026

^1^ Standard error; ^2^ clinical mastitis; ^3^ environmental pathogens: *Candida kefyr*, *Candida rugosa*, *Candida tropicalis*, *Escherichia coli*, *Enterobacter cloacae*, *Enterococcus faecalis*, *Enterococcus faecium*, *Klebsiella pneumoniae*, *Prototheca* spp., *Pseudomonas aeruginosa*, *Salmonella* sp., *Streptococcus dysgalactiae*, *Serratia marcescens*, *Streptococcus canis*, *Streptococcus gallolyticus*, *Streptococcus oralis*, *Streptococcus pluranimalium*, *Streptococcus alactolyticus*, *Streptococcus uberis*; ^4^ subclinical mastitis.

## Data Availability

The raw data of this study will be made available by the authors (corresponding author) to any qualified researcher.
